# Supplementary material for: Metal Resistant Endophytic Bacteria Reduces Cadmium, Nickel Toxicity, and Enhances Expression of Metal Stress Related Genes with Improved Growth of Oryza Sativa, via Regulating Its Antioxidant Machinery and Endogenous Hormones
Source: Plants (Basel). 2019 Sep 23;8(10):363. doi: 10.3390/plants8100363 (PMC6844085; doi:10.3390/plants8100363)
Supplement: Supplementary file 1 [file plants-08-00363-s001.pdf]

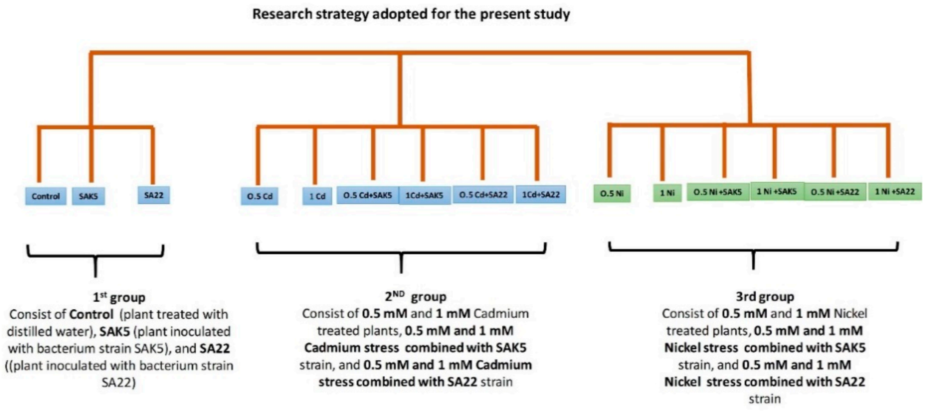

Figure S1. Research strategy adopted for the present study.

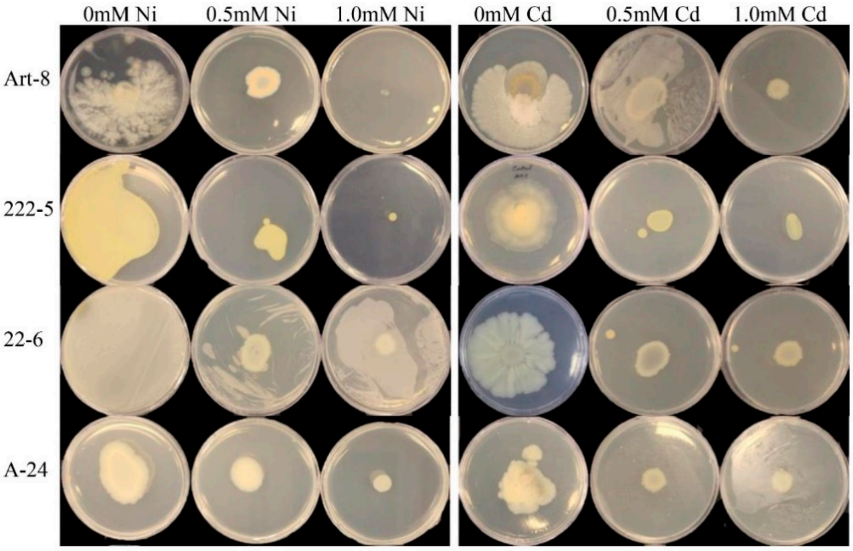

Figure S2. Screening of selected bacteria against Cd and Ni stress.

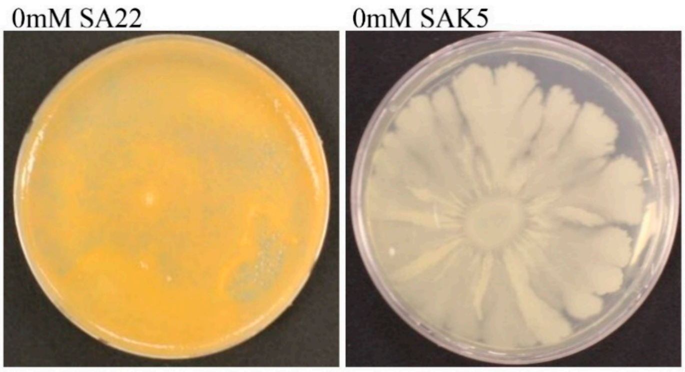

Figure S3. Determination of SA22 and SAK5 bacteria on control media (no Cd and Ni).

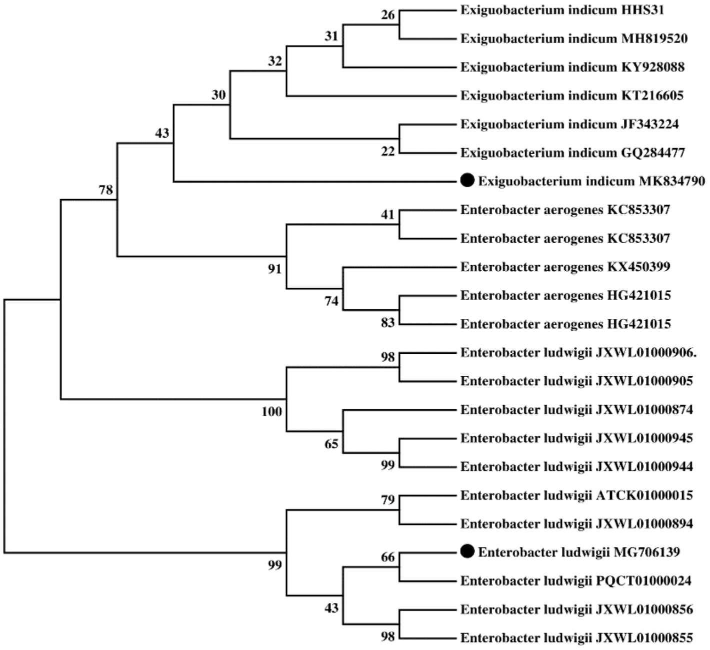

Figure S4. Molecular phylogenetic analysis of bacterial strains *Enterobacter ludwigii* SAK5 and *Exiguobacterium indicum* SA22 using neighbor-joining (NJ) method. Numbers above the branches are the NJ bootstrap values. Dot indicates *E. ludwigii* SAK5 and *E. indicum* SA22, respectively.
